# Automated Detection of Bowel Preparation Scoring and Adequacy With Deep Convolutional Neural Networks

**DOI:** 10.1093/jcag/gwac013

**Published:** 2022-04-16

**Authors:** Daniel J Low, Zhuoqiao Hong, Sechiv Jugnundan, Anjishnu Mukherjee, Samir C Grover

**Affiliations:** St. Michael’s Hospital, Toronto, ON M5B 1W8, Canada; Massachusetts Institute of Technology, Cambridge, MA 02139, USA; St. Michael’s Hospital, Toronto, ON M5B 1W8, Canada; IIEST Shibpur, Howrah, West Bengal 711103, India; St. Michael’s Hospital, Toronto, ON M5B 1W8, Canada

**Keywords:** Artificial Intelligence, Bowel Preparation, Machine Learning, Quality Indicators

## Abstract

**Introduction:**

Adequate bowel preparation is integral to effective colonoscopy. Inadequate bowel preparation has been associated with reduced adenoma detection rate and increased post-colonoscopy colorectal cancer (PCCRC). As a result, the USMSTF recommends early interval reevaluation for colonoscopies with inadequate bowel preparation. However, bowel preparation documentation is highly variable with subjective interpretation. In this study, we developed deep convolutional neural networks (DCNN) to objectively ascertain bowel preparation.

**Methods:**

Bowel preparation scores were assigned using the Boston Bowel Preparation Scale (BBPS). Bowel preparation adequacy and inadequacy were defined as BBPS ≥2 and BBPS <2, respectively. A total of 38523 images were extracted from 28 colonoscopy videos and split into 26966 images for training, 7704 for validation, and 3853 for testing. Two DCNNs were created using a Densenet-169 backbone in PyTorch library evaluating BBPS score and bowel preparation adequacy. We used Adam optimiser with an initial learning rate of 3 × 10^−4^ and a scheduler to decay the learning rate of each parameter group by 0.1 every 7 epochs along with focal loss as our criterion for both classifiers.

**Results:**

The overall accuracy for BBPS subclassification and determination of adequacy was 91% and 98%, respectively. The accuracy for BBPS 0, BBPS 1, BBPS 2, and BBPS 3 was 84%, 91%, 85%, and 96%, respectively.

**Conclusion:**

We developed DCCNs capable of assessing bowel preparation adequacy and scoring with a high degree of accuracy. However, this algorithm will require further research to assess its efficacy in real-time colonoscopy.

## Introduction

Endoscopy quality measures such as cecal intubation, withdrawal time, and bowel preparation have been associated with improved adenoma detection rate (ADR) and reduction of post-colonoscopy colorectal cancer (PCCRC) (1-6). As a result, major gastroenterology societies have quality benchmarks to ensure high-quality colonoscopy, including bowel preparation. At present, the minimum acceptable rate of adequate bowel preparation in colonoscopy is 85% (7). Adequate bowel preparation is defined as the preparation required to identify a polyp >5 mm in size (7). This is clinically relevant, as adequate bowel preparation allows for the use of recommended surveillance and screening interval targets. The USMSTF recommends repeat colonoscopy within 1 year in the event of inadequate bowel preparation (8). Moreover, high-quality bowel preparation is associated with higher ADR and detection of higher risk adenomas, including sessile serrated polyps (4,9). On the other hand, low-quality and inadequate bowel preparation is associated with higher rates of interval advanced adenomas, higher adenoma miss rates, and PCCRC (10-13).

Given the importance of bowel preparation in colonoscopy, there have been multiple evaluative scales developed to describe preparation quality. These bowel preparation scales include the Aronchik, Ottawa Bowel Preparation Scale, and the Boston Bowel Preparation Scale (BBPS) (14-16). Specifically, the BBPS has been characterized as a simple and validated tool with excellent intra and inter-observer reliability in assessing bowel preparation (4, 16). Moreover, the BBPS has been evaluated as a standardized means to characterize bowel preparation adequacy (17). Considering the impact of bowel preparation on adenoma detection, the ability to accurately and reproducibly gauge bowel preparation quality is of the utmost importance. Although current bowel preparation scales have important clinical utility, there are still inherent shortcomings within these scoring systems, including subjective interpretation (18). Further, the reporting and documentation of bowel preparation scores remains highly variable across physicians and centres and has been noted to be as low as 20% (19). Given these current limitations, there have been efforts to innovate ways to automate objective documentation of bowel preparation.

In particular, artificial intelligence (AI) has been recently applied to endoscopy quality. There have been multiple studies evaluating artificial intelligence implementation into withdrawal time, colonoscopy completion, and bowel preparation (20). However, current AI bowel preparation algorithms do not directly evaluate bowel preparation adequacy, which can inform the timing of interval colonoscopy. In this study, we developed deep convolutional neural networks capable of determining bowel preparation adequacy and bowel preparation sub-classifications scores.

## Methods

This was a retrospective study conducted at St. Michael’s Hospital in Toronto, Canada, and was approved by the St. Michael’s Hospital Research Board (19-050).

### Datasets and Preprocessing

The image database for this study was compiled using de-identified images of the colonic lumen taken from colonoscopy videos. The videos did not contain any patient identifiers and only images of colonic lumen were extracted. A total of 144 videos were collected and screened from procedures between 2015 and 2018 (21). Included videos had to be complete, which was defined as advancement of the colonoscope from rectum to cecum, and withdrawal back to the rectum. Videos not meeting these criteria were excluded. Twenty-eight videos were selected and processed into images at 10 frames per second using Adobe Photoshop CC2019 software (San Jose, California, USA). Images were assigned a Boston Bowel Preparation Scale score of 0 (segment of the mucosa not seen due to solid stool), 1 (areas of the mucosa not seen due to residual stool, staining, or opaque liquid), 2 (mucosa well seen with minor staining, residual stool, opaque liquid), or 3 (no staining, stool, or opaque liquid). Adequate bowel preparation was defined as BBPS ≥ 2, while inadequate bowel preparation was defined as BBPS < 2. Ambiguous images, such as those that were blurry or had devices present, irrigation, or fluid levels, were excluded from the dataset. Image sorting was performed by expert gastroenterologists (i.e., >1000 colonoscopies completed). Two gastroenterologists performed image sorting for all images. If there was a discrepancy between the gastroenterologists in BBPS scoring, a third gastroenterologist would decide the final BBPS score for the image.

A total of 38523 images were collected from the videos. Within this data set, 26,966 (70%) images were used for training, 7704 (20%) for validation, and 3853 (10%) for testing. The proportion of BBPS scores across each of the training, validation, and testing phases remained consistent. The testing data were held out from the model during the training and validation process. The dataset was augmented to allow for more variable conditions. Strategies that were utilized include randomized cropping, horizontal flipping, vertical flipping, affine transformations, and random rotations.

### Dense Convolutional Neural Network

We used DenseNet-169, a dense convolutional neural network architecture pretrained on 1.2 million images with SIFT transforms from the ImageNet dataset as the backbone of our model. Two neural networks were built with this architecture with the last layer replaced with a customized classifier for bowel preparation (one multi-class classifier for BBPS 0, 1, 2, and 3; and one binary classifier for BBPS <2 and ≥2). All the experiments were conducted using PyTorch library.

### Training and Testing

We used a batch size of 64 for the multi-class classifier and 128 for the binary classifier for training and validation. Adam optimizer with an initial learning rate of 3 × 10^−4^ was used along with a step scheduler. The scheduler modifies the learning rate every 7 epochs if there has been no improvement in performance to force the model towards an optimum. Focal loss function was used for loss criterion, due to its relevance for datasets with class imbalances. In training, we performed weighted sampling with replacement from a multinomial distribution to ensure that the distribution of classes within each batch was as balanced as possible to reduce bias. During validation and testing, weighted sampling was not conducted in order to ascertain the accuracy of the model on the original data distribution.

### Outcomes and Statistical Analysis

The primary outcome of the study was to evaluate the operating characteristics of two convolutional neural networks: one subclassifying Boston Bowel Preparation Scale scores and one classifying bowel preparation adequacy. The operating characteristics include accuracy, sensitivity, specificity, positive predictive value, negative predictive value, and F1 scores.

## Results

### Dataset Characteristics

There were 38523 images extracted from 28 videos. There were 381 (1.0%) BBPS0 images, 7295 (18.9%) BBPS1 images, 14,059 (36.5%) BBPS 2 images, and 16,788 (43.6%) BBPS 3 images. There were 30,847 (80.1%) adequate bowel preparation images (BBPS ≥ 2) and 7676 (19.9%) inadequate bowel preparation images (BBPS < 2).

### Bowel Preparation Classifier

A total of 3853 images were used in the test dataset for both BBPS subclassification and bowel preparation adequacy. There were 44 BBPS 0 images, 726 BBPS 1 images, 1402 BBPS 2 images, and 1681 BBPS 3 images. There were 770 inadequate BBPS scores and 3083 adequate BBPS scores. The overall accuracy of the BBPS subclassification algorithm was 91%. The accuracy of each bowel preparation subclass was 84%, 91%, 85%, and 96% for BBPS 0, BBPS1, BBPS2, and BBPS3, respectively. The operating characteristics of the model are outlined in Table 1.

**Table 1.** Detection characteristics of deep convolutional neural network for Boston Bowel Preparation Scale multi-classifier

	Accuracy	F1	PPV	NPV	Specificity	Sensitivity
BBPS 0	0.84	0.91	1.00	1.00	1.00	0.84
BBPS 1	0.91	0.92	0.92	0.98	0.98	0.91
BBPS 2	0.85	0.88	0.91	0.92	0.95	0.86
BBPS 3	0.96	0.94	0.91	0.97	0.93	0.96

### Bowel Preparation Adequacy

With regard to binary classification of bowel preparation adequacy, the overall accuracy of the model was 98%. The accuracy of the model was 91% and 98% for determination of inadequate and adequate bowel preparation, respectively. The operating characteristics of the model are outlined in Table 2.

**Table 2.** Detection characteristics of deep convolutional neural network for adequate and inadequate bowel preparation scale scores

	Accuracy	F1	PPV	NPV	Specificity	Sensitivity
BBPS <2	0.91	0.94	0.96	0.98	0.99	0.92
BBPS >2	0.98	0.98	0.98	0.96	0.92	0.99

## Discussion

In this study, we created deep convolutional neural networks capable of automating image classification into Boston Bowel Preparation Scale scores and determining bowel preparation adequacy. The overall accuracy of the BBPS score subclassifier and BBPS adequacy score algorithm was 91% and 98%, respectively. With regard to the operating characteristics of the bowel preparation adequacy algorithm, the sensitivity, specificity, PPV, and NPV exceeded 90% (Tables 3 and 4).

**Table 3.** Confusion matrix for Boston Bowel Preparation Scale multi-classifier deep convolutional neural network

	BBPS 0	BBPS 1	BBPS 2	BBPS 3
BBPS 0	37	4	3	0
BBPS 1	0	661	55	10
BBPS 2	0	52	1200	150
BBPS 3	0	1	59	1621

**Table 4.** Confusion matrix for bowel preparation adequacy convolutional neural network

	BBPS <2	BBPS >2
BBPS < 2	708	62
BBPS >2	32	3051

The model performed exceptionally well at determining bowel preparation adequacy. Despite this, notable class imbalance between BBPS≥ 2 and BBPS<2 existed. Although similar techniques with weighted sampling were used to train the deep convolutional networks in both BBPS scoring and bowel preparation adequacy, the class imbalances were greater in the BBPS subclassification model leading to slightly inferior operating characteristics. For instance, there were only 381 BBPS 0 images, representing <1% of the total dataset. This translated to significantly less variability of training images compared to any other class despite modified sampling strategies. As a result of the restricted dataset, the generalizability of the bowel preparation subclassification model for BBPS 0 is limited. On the other hand, in terms of the bowel preparation adequacy algorithm, class imbalances were reduced, with inadequate bowel preparation images (BBPS0 and BBPS1) representing approximately 20% of the total dataset. Despite the class disparity in the bowel preparation adequacy model, there was excellent discrimination between the two classes with high accuracy. In this case, the aggregate BBPS 0 and BBPS 1 scores were sufficiently variable to represent the inadequate bowel preparation class. Ultimately, to improve our BBPS subclassification model, we require a larger number of BBPS 0 images to balance the dataset to improve variability and generalizability.

With regard to BBPS 2 images in the BBPS score subclassifier, the operating characteristics were slightly inferior compared to other classes. The majority of the misclassified BBPS2 images were sorted as BBPS 3 images. This is likely related to our strict sorting criteria when creating the image library, wherein small residual stains were classified as BBPS 2. A more liberal interpretation would have correctly identified these images as BBPS 3. However, in terms of clinical relevance, adenoma detection is similar between BBPS 2 and BBPS 3, and BBPS 2 bowel preparation is sufficient for adenoma detection >5 mm (17, 21). In order to improve the BBPS 2 operating characteristics, the sorting criteria will need to be liberalized, but the clinical relevance of more accurately differentiating BBPS 2 from BBPS 3 is possibly limited. More importantly, the current bowel preparation adequacy algorithm has excellent operating characteristics and can guide decisions around interval colonoscopy. However, this algorithm was trained, validated, and tested on images taken from insertion and withdrawal which is not representative of current clinical practice in ascertaining bowel preparation. As such, further validation studies need to be conducted in real-time on withdrawal in a more clinically relevant manner.

In our review of the literature, there have been prior attempts in applying artificial intelligence to bowel preparation. Su et al. developed a quality system which assessed BBPS score as a continuous variable, and extrapolated adequacy if BBPS scores were greater than 2 (22). On the other hand, Zhou et al. automated detection and sorting of images to discrete BBPS scores (23). Our algorithm adds to the current literature by doubly assessing bowel preparation adequacy and BBPS subclassification. The addition of a dedicated algorithm to assess for adequacy, rather than extrapolating adequacy from BBPS scores, has possibly greater validity compared to prior attempts. The ability to automate detection of bowel preparation adequacy has significant clinical utility. Critically, inadequate bowel preparation is associated with higher interval rates of advanced adenomas and PCCRC (24). As a result, colonoscopies with inadequate bowel preparation scale scores should undergo early endoscopic reassessment. Moreover, adequate bowel preparation determines whether routine surveillance recommendations can be advised. As such, a dedicated bowel preparation adequacy algorithm can be applied to assist in best practice decision making for interval endoscopic evaluation. Despite the accuracy of our bowel preparation adequacy algorithm, additional research and integration into real-time colonoscopy is required for further assessment.

The development and implementation of utilizing a standardized algorithm to automatically assess the quality of bowel preparation in the clinical setting has impactful implications. In clinical practice, bowel preparation documentation is highly variable, reaching rates as low as 20% (19). Given the importance of adequate bowel preparation in ensuring effective colonoscopy, it is critical to have consistent determination and documentation of bowel preparation quality. However, manual bowel preparation scores assigned by endoscopists are subject to inherent subjectivity (18). Subjectivity may be influenced by factors such as baseline differences in experience or interpretation between endoscopists. For example, the BBPS, which is highly validated and widely used, has substantial inter-rater variability with an inter-rater coefficient often around 0.71 (25, 26). Utilization of an automated bowel preparation algorithm in real-time would eliminate this bias. When compared to pre-existing bowel preparation scores that rely on segments of bowel or a gestalt of the complete examination, automated scoring systems may provide a more accurate and detailed assessment of bowel preparation in real-time. As a result, a bowel preparation artificial intelligence algorithm may improve upon limitations in bowel preparation scales and in clinical practice documentation.

In summary, deep convolutional neural networks were developed to automate bowel preparation score subclassification and to determine bowel preparation adequacy with excellent accuracy. Moving forward, the algorithm will require a larger balanced dataset to improve accuracy and will need to be validated in real-time colonoscopy. The role of automated quality indicator algorithms in clinical practice requires further research and evaluation but has significant theoretical benefits.
